# Draft Genome Sequences of Six Nontypeable *Haemophilus influenzae* Strains That Establish Bacteremia in the Infant Rat Model of Invasive Disease

**DOI:** 10.1128/genomeA.00899-15

**Published:** 2015-09-24

**Authors:** Timothy M. VanWagoner, Daniel J. Morton, Thomas W. Seale, Huda J. Mussa, Brett K. Cole, Paul W. Whitby, Terrence L. Stull

**Affiliations:** a Department of Pediatrics, University of Oklahoma Health Sciences Center, Oklahoma City, Oklahoma, USA; b Department of Microbiology and Immunology, University of Oklahoma Health Sciences Center, Oklahoma City, Oklahoma, USA

## Abstract

*Haemophilus influenzae* is an important cause of invasive disease. The infant rat is the accepted model of invasive *H. influenzae* disease. Here, we report the genome sequences of six nontypeable *H. influenzae* strains that establish bacteremia in the infant rat.

## GENOME ANNOUNCEMENT

*Haemophilus influenzae* colonizes the human nasopharynx and is an opportunistic pathogen causing significant human disease. *H. influenzae* causes both mucosal and invasive infections, including otitis media, exacerbations of chronic obstructive pulmonary disease, and bacteremia (1–3). Vaccines directed at the *H. influenzae* type b capsule have significantly reduced invasive disease caused by such strains (3), and the majority of *H. influenzae* invasive disease is now caused by nontypeable strains (1). The infant rat is a widely used model of invasive *H. influenzae* disease (4, 5). *H. influenzae* type b strains readily establish bacteremia and meningitis in this model when infected via either the intraperitoneal or nasopharyngeal route (6). However, nontypeable *H. influenzae* (NTHi) strains less reliably establish and maintain bacteremia in this model. We have previously used the sequenced blood isolate R2866 in the rat model (7). An infective dose of 10^5^ CFU R2866 reproducibly establishes durable bacteremia. However, in our hands, other well-characterized NTHi strains isolated from the nasopharynx (strain 86-028NP) and middle ear (strain R2846) do not establish bacteremia, even at infective doses up to 10^6^ CFU. For ongoing studies on NTHi virulence we required additional strains able to establish bacteremia in the infant rat. We tested 14 NTHi isolates, all isolated from blood, CSF, or brain abscess, for their ability to establish bacteremia in the infant rat. Six strains were able to reproducibly establish bacteremia in the infant rat, while the rest were unable to do so regardless of dose.

We performed whole-genome sequencing on the six isolates that established bacteremia. NTHi samples were prepared using 50 ng of total genomic DNA according to Nextera DNA library kit protocols (Illumina, Inc). Samples were indexed according to standard protocols so that they could be pooled together and sequenced simultaneously in a single run on the Illumina MiSeq platform using paired-end 150-bp (strains HI1974, HI2114, and HI2116) or paired-end 250-bp (strains HI1980, HI1988, and HI2004) chemistry. Prior to sequencing, each library was run individually on the Agilent High Sensitivity DNA chip to confirm library quality and average insert size. Samples were pooled in equimolar amounts, and 8 pM of the pool was run on the sequencer. Per Illumina’s recommendation, phiX control was spiked into the library pool prior to loading for quality control purposes. For the 300-cycle runs 5 to 10% phiX was used, and for the 500-cycle runs 1% phiX was used per Illumina protocols. A total of thirty to forty million reads were collected for each run. Raw sequence data were *de novo* assembled and aligned to a reference isolate using CLC Genomics Workbench (CLC Bio) to identify single-nucleotide polymorphisms as well as regions with insertions or deletions (indels). Sequencing results are summarized in Table 1. The sequences reported herein will facilitate further *in vivo* studies of invasive NTHi disease and virulence determinants.

**TABLE 1:** Summary of genome sequences for six invasive NTHi isolates

Strain name	Source	BioSample no.	GenBank accession no.	Genome coverage (×)	No. of contigs	G+C content (%)	Genome size (Mb)	No. of genes
HI1974	Blood	SAMN03702706	LFFT00000000	348	41	37.9	1.77	1,739
HI1980	Blood	SAMN03702713	LFFO00000000	1,088	20	37.8	1.76	1,712
HI1988	Blood	SAMN03702714	LFFN00000000	978	18	37.9	1.84	1,814
HI2004	Blood	SAMN03702711	LFFQ00000000	1,052	21	37.9	1.77	1,736
HI2114	Blood	SAMN03702707	LFFR00000000	816	75	37.9	1.89	1,865
HI2116	Blood	SAMN03702708	LFFS00000000	387	34	38.0	1.83	1,793

### Nucleotide sequence accession numbers.

Genome sequences for the six strains described herein have been deposited in GenBank under the accession numbers listed in Table 1.

## References

[1] Van Eldere J, Slack MP, Ladhani S, Cripps AW 2014 Non-typeable *Haemophilus influenzae*, an under-recognised pathogen. Lancet Infect Dis 14:1281–1292. doi:10.1016/S1473-3099(14)70734-0. 25012226

[2] Alikhan MM, Lee FE 2014 Understanding nontypeable *Haemophilus influenzae* and chronic obstructive pulmonary disease. Curr Opin Pulm Med 20:159–164. doi:10.1097/MCP.0000000000000023. 24441573

[3] Ladhani SN 2012 Two decades of experience with the *Haemophilus influenzae* serotype b conjugate vaccine in the United Kingdom. Clin Ther 34:385–399. doi:10.1016/j.clinthera.2011.11.027. 22244051

[4] Smith AL, Smith DH, Averill DR, Marino J, Moxon ER 1973 Production of *Haemophilus influenzae* B meningitis in infant rats by intraperitoneal inoculation. Infect Immun 8:278–290. 4542033 10.1128/iai.8.2.278-290.1973 PMC422844

[5] Seale TW, Morton DJ, Whitby PW, Wolf R, Kosanke SD, VanWagoner TM, Stull TL 2006 Complex role of hemoglobin and hemoglobin-haptoglobin binding proteins in *Haemophilus influenzae* virulence in the infant rat model of invasive infection. Infect Immun 74:6213–6225. doi:10.1128/IAI.00744-06. 16966415 PMC1695506

[6] Moxon ER, Glode MP, Sutton A, Robbins JB 1977 The infant rat as a model of bacterial meningitis. J Infect Dis 136(Suppl):S186–S190. 330777 10.1093/infdis/136.supplement.s186

[7] Hempel RJ, Morton DJ, Seale TW, Whitby PW, Stull TL 2013 The role of the RNA chaperone Hfq in *Haemophilus influenzae* pathogenesis. BMC Microbiol 13:134. doi:10.1186/1471-2180-13-134. 23767779 PMC3691723

